# Opioid Use and Storage Patterns by Patients after Hospital Discharge following Surgery

**DOI:** 10.1371/journal.pone.0147972

**Published:** 2016-01-29

**Authors:** Karsten Bartels, Lena M. Mayes, Colleen Dingmann, Kenneth J. Bullard, Christian J. Hopfer, Ingrid A. Binswanger

**Affiliations:** 1 Department of Anesthesiology, University of Colorado Denver, Aurora, Colorado, United States of America; 2 Department of Anesthesiology, Children's Hospital Colorado, Aurora, Colorado, United States of America; 3 Division of Substance Dependence, Department of Psychiatry, University of Colorado Denver, Aurora, Colorado, United States of America; 4 Institute for Health Research, Kaiser Permanente Colorado, Denver, Colorado, United States of America; 5 Division of General Internal Medicine, Department of Medicine, University of Colorado Denver, Aurora, Colorado, United States of America; Boston Children’s Hospital and Harvard Medical School, UNITED STATES

## Abstract

**Introduction:**

Opioid-based analgesic therapy represents a cornerstone of pain management after surgery. The recent rise in opioid sales and opioid overdoses suggests it is important to maximize the safety of opioid prescribing after surgery. Given that patients may live with other family members in the home, safe storage and appropriate disposal of excess opioids after hospital discharge are necessary to prevent unintended secondary exposures. Identifying characteristics of patients who are likely to be prescribed excess opioids after surgery may enable more targeted prescription practices and safety interventions. Our study aimed to elucidate patient-reported opioid use patterns and modes of home storage of opioids among patients discharged home after Cesarean section (C-section) and thoracic surgery. Specifically, we sought to identify characteristics of patients who reported using about half or more versus less of the opioids prescribed to them for use after hospital discharge.

**Methods:**

For this cohort study, we developed a survey on quality of analgesia following hospital discharge, amounts of opioids taken relative to the amount prescribed, reasons for not taking all prescribed medications, and storage and disposal methods for leftover opioids. Adult patients, who had C-section or thoracic surgery at a tertiary academic medical center, were given a web-based self-administered survey after discharge. Descriptive statistics (means and standard deviations, proportions) were used to describe the study sample and survey results. Comparisons between patients who reported taking about half or more versus less of the opioids prescribed to them for use after hospital discharge were made using unpaired t-tests, Mann-Whitney tests, and Chi-square tests as appropriate.

**Results:**

The majority (53%) of respondents after C-section (N = 30) reported taking either no or very few (less than 5) prescribed opioid pills; 83% reported taking half or less; and 17% of women, reported taking all or nearly all (5 or fewer pills left over) of their opioid prescription. In a cohort of patients after thoracic surgery (n = 31) 45% reported taking either no or very few (5 or less) prescribed opioid pills; 71% reported taking half or less; and 29% of patients reported taking all or nearly all (5 or fewer pills left over) of their opioid prescription. In both cohorts, use of opioids while hospitalized was higher in the group reporting using about half or more of prescribed opioids after discharge. Leftover opioids were stored in an unlocked location in 77% and 73% of cases following C-section and thoracic surgery, respectively.

**Conclusion:**

Our findings from surveys in two distinct patient populations at a single academic medical center suggest that current opioid prescribing practices for pain management at hospital discharge following Cesarean section and thoracic surgery may not account for individual patients’ analgesic requirements. Excess opioid pills are commonly stored in unsecured locations and represent a potential source for non-medical opioid use and associated morbidity and mortality in patients and their families. Research to develop goal-directed and patient-centered post-discharge opioid prescription practices and encourage opioid safety practices after surgery is needed.

## Introduction

Compassionate and effective post-surgical pain therapy forms a cornerstone of perioperative care. Since pain was coined “the fifth vital sign” in the 1990s, sales of prescription opioids in the Unites States have quadrupled [1]. This increase has been accompanied by a dramatic rise in prescription opioid-associated morbidity and mortality [2]. In 2010, more than 16,000 deaths were attributed to prescription opioids [3], making them a leading cause of injury death in the general population [4]. Among women, opioid overdose deaths have risen five-fold from 1999–2010 [5]. Cesarean section (C-section) is the most common surgery performed in the United States [6], and women are commonly prescribed opioids after C-section. After surgery, unsecured prescription opioids may represent a particular danger in a home with young children [7, 8]. Limiting access to unused medications while ensuring effective pain management has been proposed as a strategy for improving current prescription practices [9]. Identifying patients who are unlikely to use most of their prescribed opioid medications could enable clinicians to safely prescribe fewer opioid pills after surgery. Patients who report using less than half of the prescribed medications would constitute a promising target for individualized and goal-directed opioid prescriptions that would reduce over-prescription, without compromising adequate analgesia.

After minor surgical procedures patients do not take many of the opioids prescribed to them [10–12], but less is known about use patterns after major surgery such as C-section and thoracic surgery. There is a paucity of information about how patients store unused opioids, but a small study of patients following emergency room visits indicated that safe storage and proper disposal rarely occurs [13]. As the initial step to improve post-hospital discharge pain management practices, we developed and tested a survey to elucidate current usage patterns of prescribed opioids among women after C-section surgery. We hypothesized that the majority of patients would not require all of the opioids prescribed to them. In addition, we sought to describe reasons for not taking all prescribed opioids and elucidate patterns for storage and disposal for unused opioids. To confirm our findings, we then chose to survey a second, distinct cohort of surgical patients in which use and storage patterns may be different. Thoracic surgery is associated with high levels of pain and an elevated incidence of long-term opioid use after surgery [14]. Given that C-section represents a relatively uniform surgery with comparable tissue injury in a homogenous patient population, thoracic surgery patients have variable patient characteristics and postoperative pain levels [15]. Hence, this cohort represents a suitable population for comparison.

## Methods

We conducted a survey of patients discharged from the hospital after C-section or thoracic surgery. This study was approved by the Colorado Multiple Institutional Review Board (COMIRB), protocol # 14–1938. Potential participants were identified by screening the records of patients who underwent C-section at the University of Colorado Hospital from November 2014 –January 2015 for eligibility. The electronic medical record was searched on a weekly basis for C-section procedures. Patients returning to institutional settings, younger than 18 years of age, and unable to speak English or Spanish were excluded. A follow-up cohort of patients who had thoracic surgery at the University of Colorado Hospital from August 2015 –November 2015 was identified in similar fashion.

### Survey Instrument

**Fig 1:**
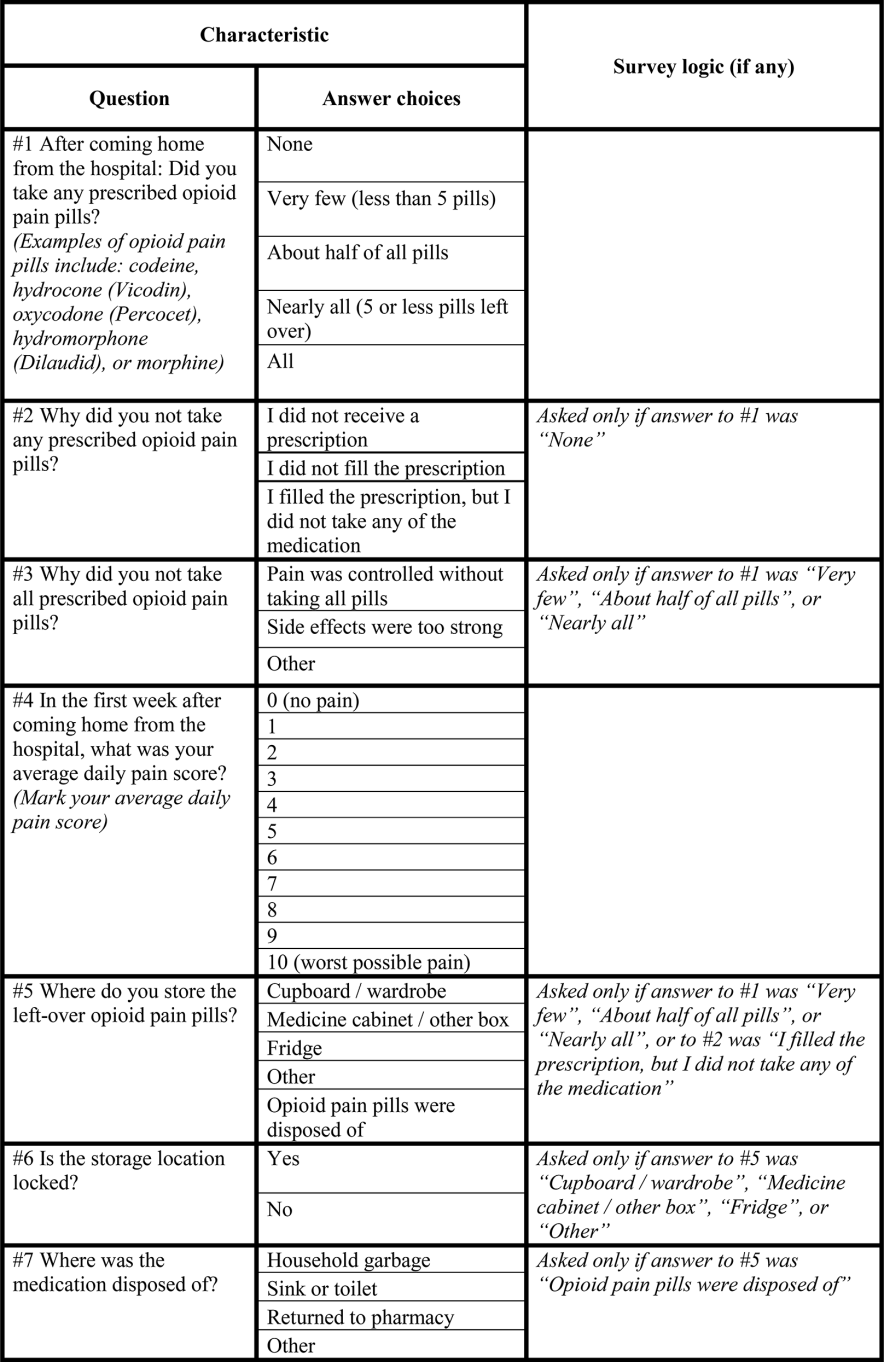
Survey tool administered to patients discharged after Cesarean section surgery.

Due to limited prior research on post-surgical use and storage of opioids, previously validated survey tools were unavailable for use in this study. Thus, study investigators developed a survey instrument with input from experts in post-operative pain management as well as opioid use [16, 17]. The survey domains included the following: quality of analgesia following hospital discharge, amounts of opioids taken relative to the amount prescribed, reasons for not taking all prescribed medications, and storage and disposal methods for leftover opioids. Readability was assessed with a desired Flesch-Kincaid Grade level score of 8 or lower [18]. Surveys were made available in Spanish and English language. A certified medical translator performed the initial translation. A second back-translation from Spanish to English (blinded to the English original) was performed to ensure the quality of the final translation used in the survey. Face validity was assessed by circulating the survey amongst individuals outside of the study team, which led to revisions in the final survey instrument (Fig 1). For the thoracic surgery cohort, an amended survey was used (S2 Table).

### Participant Recruitment and Consent

Survey invitations were sent via email or postal mail. Emails included a link to the consent form and study, whereas mailed letters asked for the email address to be provided and subsequently the survey would be administered electronically. We sent up to two follow-up reminders. The introductory letter informed patients how the results of the survey could improve future pain therapy, as recommended by Groves et al. to encourage participation [17, 19]. The Principal Investigator’s contact information was also shared in the introductory letter to enable direct communication and display positive regard for the participants [20]. In addition, we thanked readers of the introductory letter for their time, which has also been associated with increased likelihood of responding to a survey [21]. Patients were consented for the study using electronic postcard format prior to taking the survey.

The introductory letter also introduced a U.S. $ 10 compensation to complete the C-section survey. To improve response rate, we increased this amount to U.S. $ 25 for the survey in the thoracic surgery cohort [22]. Data from the self-administered web-based survey was collected electronically using Research Electronic Data Capture (REDCap™), a secure, HIPAA-compliant web-based application designed for data collection in research studies [23]. Baseline characteristics of the study population were collected from the medical record. Opioid analgesic doses administered to patients in the hospital in the 24 hours prior to discharge as well as prescriptions for post-discharge use were abstracted from the medical record and converted to oral morphine equivalents, accounting for the distinctive potency of different opioids [24–27].

To characterize patients who would likely have their pain successfully managed with lower doses of post-discharge opioids, we dichotomized survey responders into patients reporting to have used none or very few of the prescribed opioids *versus* patients who reported taking about half or more of what was prescribed to them. Descriptive statistics (means, standard deviations, proportions) were used to describe the study sample and survey results. Comparisons between categorical data were made using Chi-square test. Distribution of continuous data was assessed using the D'Agostino & Pearson omnibus normality test. Comparisons between continuous variables amongst two groups were made using unpaired t-test for normally distributed and Mann-Whitney test for non-normally distributed data. Graph Pad Prism 6.0 (GraphPad Software Inc., La Jolla, CA, U.S.A.) was used for all statistical analysis.

## Results

The post C-section survey invitation was sent to 116 patients. Thirty patients completed the survey (response rate 26%). The post thoracic surgery survey was sent to 107 patients. Thirty-three patients participated in the survey (response rate 31%). One patient responded that he wanted to participate in the survey, but did not complete any further questions and, therefore, was excluded from analysis. Another survey was returned from a patient that did not speak English or Spanish and was also excluded. Average duration (standard deviation in brackets) from hospital discharge to return of the survey was 30 (12) days for C-section patients and 32 (14) days for thoracic surgery patients.

**Fig 2:**
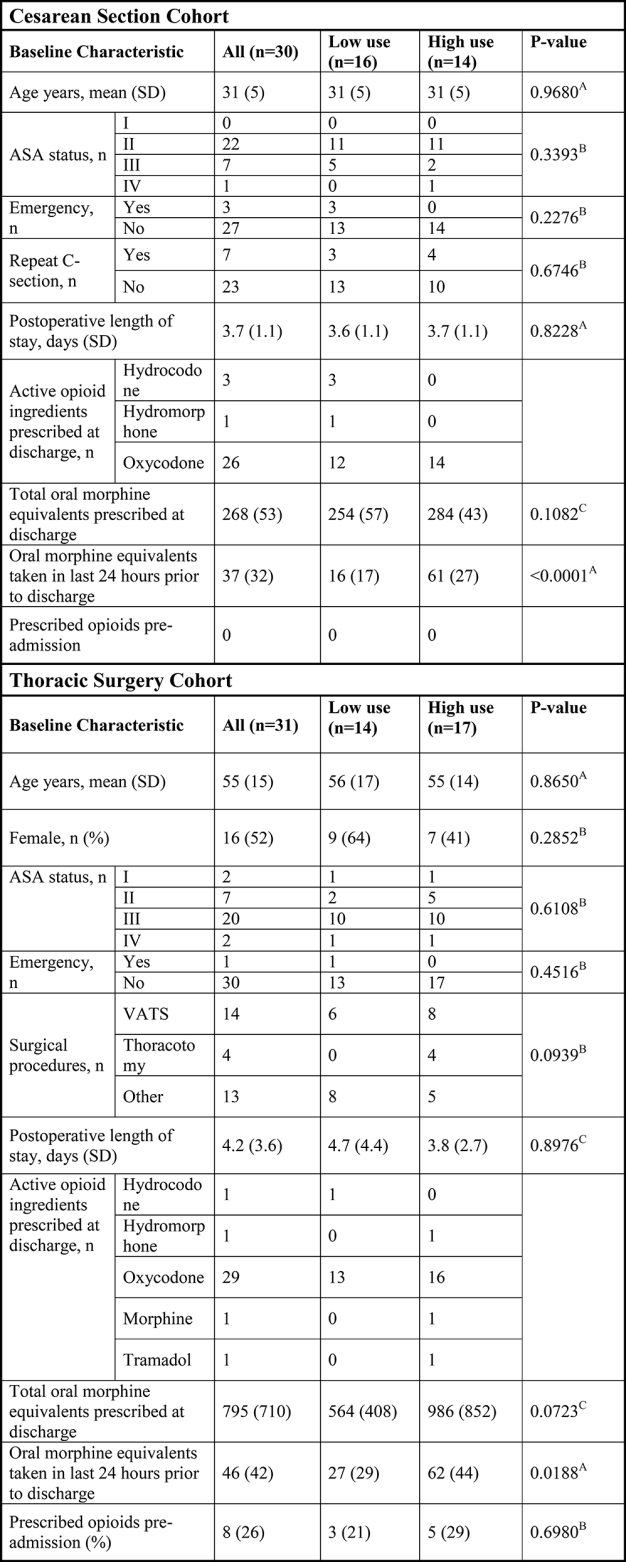
Baseline characteristics of survey responders and by reported use of prescribed opioids after discharge. Oral morphine equivalent doses are expressed in mg: oxycodone (mg) x 1.5; hydrocodone (mg) x 1; hydromorphone (mg) x 4, morphine (mg) x 1, and tramadol (mg) x 0.2. In the thoracic surgery group, two patients received more than one active opioid ingredient. “Low use” was defined as having reported taking less than half of their prescribed opioid pain medications (question #1 in the survey). “High use” was defined as having reported taking about half or more of the opioids prescribed. Statistical analysis was performed using unpaired t test^A^, Chi-square test^B^, and Mann-Whitney test^C^ between patients reporting “low use” and “high use”. For the characteristic “Active opioid ingredients prescribed at discharge” multiple opioids could be prescribed for one patient; no statistical comparisons were performed. ASA = American Society of Anesthesiologists; VATS = video-assisted thoracoscopic surgery.

**Fig 3:**
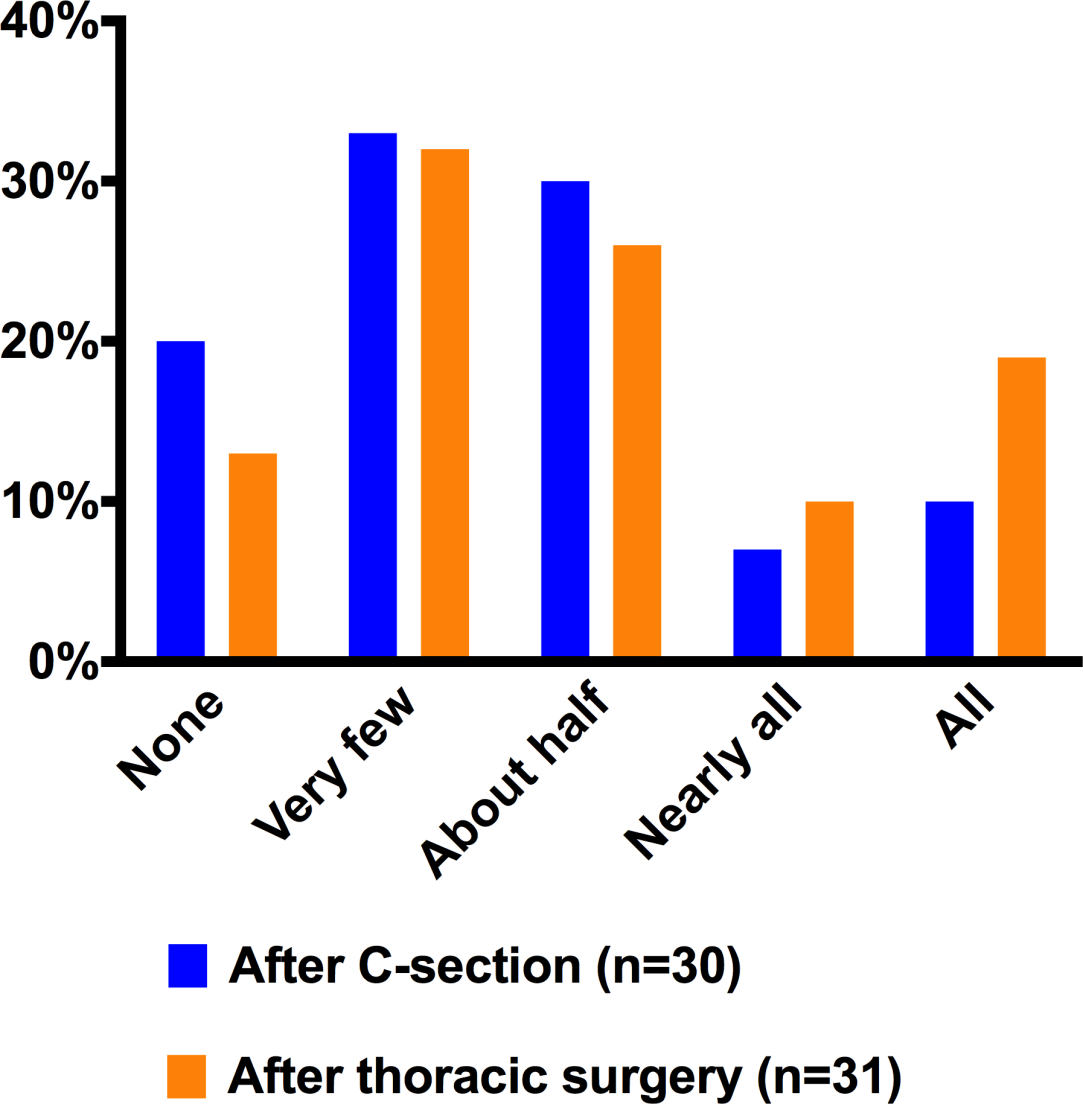
Self-reported opioid intake after surgery. C-section patients were asked: “After coming home from the hospital: Did you take any prescribed opioid pain pills?” Thoracic surgery patients were asked: “After coming home from the hospital: How many prescribed opioid pain pills did you take?” Additional details on survey questions and responses are reported in S1 and S2 Tables.

Baseline characteristics of survey respondents are given in Fig 2. The majority, 53% of respondents after C-section, reported taking either none or very few of the prescribed opioid pills (Fig 3). Similarly, 45% of patients discharged home after thoracic surgery reported taking either none or very few of prescribed opioids (Fig 3). We dichotomized the survey responders into two groups of “low-use” and “high use”, indicating whether they responded to taking very few or less versus about half or more of opioids prescribed to them for use after discharge. Patients who had higher opioid use while hospitalized (as recorded in the medical record), were more likely to report higher use of opioids after discharge. Mirroring these findings, low in-hospital intake was associated with low use post-discharge (Fig 2). Leftover opioids were stored in an unlocked location in 77% and 73% of cases following C-section and thoracic surgery, respectively. Detailed responses to all survey questions are reported in the Supporting Information (S1 and S2 Tables).

## Discussion

Our findings suggest that excess opioids are prescribed to the majority of C-section patients at hospital discharge in a single academic medical center in the Unites States. Eighty-three percent reported taking only half or less or their prescribed opioids. More than half of patients reported taking none or very few of the opioid pain pills prescribed to them. Survey findings in a second patient cohort after thoracic surgery revealed 45% taking none or very few of prescribed opioids and 71% taking half or less of what was prescribed.

In both patient cohorts, the primary reason for not taking all prescribed opioids was that they were not needed to achieve adequate pain control. Consistent with a “one-size-fits-all” approach to choosing a post-discharge opioid-based pain regimen, clinicians tended to over-prescribe pain medications after surgery. While this has been previously reported for patients undergoing upper extremity surgery [10], urologic [12], and dermatologic procedures [11], our study raises important issues for patients after C-section and after thoracic surgery. Our finding that leftover opioids were stored in an unsecured location in 77% (C-section) and 73% (thoracic surgery) of cases suggests a potential source for non-medical use, as well as unintentional ingestion, especially in households with a presence of infants and young children. On the other hand, given that a subset of patients (17% after C-section and 29% after thoracic surgery), took nearly all or all of their prescribed opioids, great care must be taken not to interpret our findings so that patients in need of higher total doses receive inadequate amounts of analgesic medications for use after discharge. As shown in Fig 2, patients received similar amounts of prescribed opioids, regardless of the amounts of self-reported use after discharge (there was a trend for higher total doses prescribed in the high-use thoracic surgery patients, but it failed to reach statistical significance). Since in-hospital opioid intake was higher in the post-discharge high use group and lower in the post-discharge low-use group, this may indicate an opportunity to build a predictive model. Such a model would require confirmation of our findings in a larger sample size and could allow clinicians to more precisely anticipate actual patient use after discharge. By means of an individualized approach to pain management after surgery, over- *and* under-prescription of post-discharge opioids following surgery could possibly be avoided.

Our study has several limitations. First, our response rate was 26% for the post C-section and 31% for the post thoracic surgery patients. While respondents may have differed from non-respondents in important ways, our response rate is close to results from a meta-analysis on online and web-based surveys which noted that average complete response rates were 34.6% [28]. Future surveys could be re-designed to enroll patients while they are still in the hospital to improve survey response rates. Second, our patients may have had recall bias when remembering how many opioid pain pills they took. We sent out the surveys as soon as completion of opioid pain medication consumption could have been expected in order to minimize this source of bias. And, our results are consistent with what has been found for minor surgeries [10–12]. Third, we collected data on patient-reported medication intake, which may not reflect actual intake. While alternative technologies such as the Medication Event Monitoring System have been described to successfully monitor scheduled medication adherence [29], their use for medications taken “as needed” is not yet proven. However, this may be an approach worthy of testing in future studies. In addition, our survey has not been formally validated, and should be tested in a larger sample and other surgical populations. Lastly, our study is based on a relatively small sample size (n = 61) from a single institution, which limits generalizability of our results to other surgical procedures and environments.

## Conclusions

In a survey at a United States academic medical center current opioid prescribing practices for pain management after C-section and thoracic surgery were not individualized to patient needs. This can lead to significant amounts of under- and over-prescribed opioids to patients for use after hospital discharge. Opioids stored in unsecured locations represent a potential source for non-medical opioid use and associated morbidity and mortality in patients and their families. More research to develop safer and more effective pain management strategies using patient-specific opioid prescription tools that aim to predict actual need for analgesia in patients after surgery is needed.

## Supporting Information

S1 Table Survey questions and responses from women after hospital discharge following Cesarean section (n = 30). (DOCX)

S2 Table Survey questions and responses from patients after hospital discharge following thoracic surgery (n = 31). One patient indicated that he did not take any opioid medications in the free text box of the survey. For this patient question #1 was entered as “None”.
(DOCX)
