# Supplementary material for: Opioid Use and Storage Patterns by Patients after Hospital Discharge following Surgery
Source: PLoS One. 2016 Jan 29;11(1):e0147972. doi: 10.1371/journal.pone.0147972 (PMC4732746; doi:10.1371/journal.pone.0147972)
Supplement: S1 Table — (DOCX) [file pone.0147972.s001.docx]

**S1 Table:** Survey questions and responses from women after discharge to home following Cesarean section (n=30)

| **Characteristic** | | **N (%)** |
| --- | --- | --- |
| **Question** | **Answer choices** |  |
| #1 After coming home from the hospital: Did you take any prescribed opioid pain pills?  (Examples of opioid pain pills include: codeine,  hydrocone (Vicodin), oxycodone (Percocet),  hydromorphone (Dilaudid), or morphine) | None | 6 (20) |
|  | Very few (less than 5 pills) | 10 (33.3) |
|  | About half of all pills | 9 (30) |
|  | Nearly all (5 or less pills left over) | 2 (6.7) |
|  | All | 3 (10) |
| #2 Why did you not take any prescribed opioid pain pills? | I did not receive a prescription | 0 (0) |
|  | I did not fill the prescription | 4 (66.7) |
|  | I filled the prescription, but I did not take any of the medication | 2 (33.3) |
| #3 Why did you not take all prescribed opioid pain pills? | Pain was controlled without taking all pills | 19 (82.6) |
|  | Side effects were too strong | 4 (17.4) |
|  | Other | 4 (17.4) |
| #4 In the first week after coming home from the  hospital, what was your average daily pain score?  (Mark your average daily pain score) | 0 (no pain) | 0 (0) |
|  | 1 | 1 (3.3) |
|  | 2 | 1 (3.3) |
|  | 3 | 8 (26.7) |
|  | 4 | 7 (23.3) |
|  | 5 | 6 (20) |
|  | 6 | 1 (3.3) |
|  | 7 | 4 (13.3) |
|  | 8 | 1 (3.3) |
|  | 9 | 1 (3.3) |
|  | 10 (worst possible pain) | 0 (0) |
| #5 Where do you store the left-over opioid pain pills? | Cupboard / wardrobe | 6 (26.1) |
|  | Medicine cabinet / other box | 16 (69.6) |
|  | Fridge | 0 (0) |
|  | Other | 0 (0) |
|  | Opioid pain pills were disposed of | 1 (4.3) |
| #6 Is the storage location locked? | Yes | 5 (22.7) |
|  | No | 17 (77.3) |
| #7 Where was the medication disposed of? | Household garbage | 0 (0) |
|  | Sink or toilet | 0 (0) |
|  | Returned to pharmacy | 1 (100) |
|  | Other | 0 (0) |
