# Supplementary material for: Opioid Use and Storage Patterns by Patients after Hospital Discharge following Surgery
Source: PLoS One. 2016 Jan 29;11(1):e0147972. doi: 10.1371/journal.pone.0147972 (PMC4732746; doi:10.1371/journal.pone.0147972)
Supplement: S2 Table — One patient indicated that he did not take any opioid medications in the free text box of the survey. For this patient question #1 was entered as “None”. (DOCX) [file pone.0147972.s002.docx]

**S2 Table:** Survey questions and responses from patients after discharge to home following thoracic surgery (n=31). One patient indicated that he did not take any opioid medications in the free text box of the survey. For this patient question #1 was entered as “None”.

| **Characteristic** | | **N (%)** |
| --- | --- | --- |
| **Question** | **Answer choices** |  |
| #1 After coming home from the hospital: How many prescribed opioid pain pills did you take?  (Examples of opioid pain pills include: codeine,  hydrocone (Vicodin), oxycodone (Percocet),  hydromorphone (Dilaudid), or morphine) | None | 4 (12.9) |
|  | Very few (5 or less pills taken) | 10 (32.3) |
|  | About half of all pills | 8 (25.8) |
|  | Nearly all (5 or less pills left over) | 3 (9.7) |
|  | All | 6 (19.4) |
| #2 Why did you not take any prescribed opioid pain pills? | I did not receive a prescription | 0 (0) |
|  | I did not fill the prescription | 0 (0) |
|  | I filled the prescription, but I did not take any of the medication | 3 (100) |
| #3 Why did you not take all prescribed opioid pain pills (check all that apply)? | Pain was controlled without taking all pills | 17 (70.8) |
|  | Side effects were too strong | 7 (29.2) |
|  | Concern for becoming addicted | 2 (8.3) |
|  | Other | 4 (16.7) |
| #4 In the first week after coming home from the  hospital, what was your average daily pain score? (Mark your average daily pain score) | 0 (no pain) | 1 (3.2) |
|  | 1 | 3 (9.7) |
|  | 2 | 3 (9.7) |
|  | 3 | 7 (22.6) |
|  | 4 | 7 (22.6) |
|  | 5 | 2 (6.5) |
|  | 6 | 2 (6.5) |
|  | 7 | 3 (9.7) |
|  | 8 | 2 (6.5) |
|  | 9 | 1 (3.2) |
|  | 10 (worst possible pain) | 0 (0) |
| #5 Where do you store the left-over opioid pain pills? | Cupboard / wardrobe | 5 (20.8) |
|  | Medicine cabinet / other box | 13 (54.2) |
|  | Fridge | 0 (0) |
|  | Other | 4 (16.7) |
|  | Opioid pain pills were disposed of | 2 (8.3) |
|  | I do not have any left-over opioid pain pills | 0 (0) |
| #6 Is the storage location locked? | Yes | 6 (27.3) |
|  | No | 16 (72.7) |
| #7 Where was the medication disposed of? | Household garbage | 0 () |
|  | Sink or toilet | 0 () |
|  | Returned to pharmacy | 1 (50) |
|  | Other | 1 (50) |
